# Is DWI/ADC a Useful Tool in the Characterization of Focal Hepatic Lesions Suspected of Malignancy?

**DOI:** 10.1371/journal.pone.0101944

**Published:** 2014-07-15

**Authors:** Maria Luiza Testa, Rubens Chojniak, Letícia Silva Sene, Aline Santos Damascena, Marcos Duarte Guimarães, Janio Szklaruk, Edson Marchiori

**Affiliations:** 1 Department of Diagnostic Radiology, A.C. Camargo Cancer Center, São Paulo, SP, Brazil; 2 CIPE - International Center for Research, A.C. Camargo Cancer Center, São Paulo, SP, Brazil; 3 Department of Diagnostic Radiology, The University of Texas - MD Anderson Cancer Center, Houston, Texas, United States of America; 4 Department of Radiology, Federal University of Rio de Janeiro, Petrópolis, Rio de Janeiro, Brazil; West German Cancer Center, Germany

## Abstract

**Objective:**

Apparent diffusion coefficient (ADC) values calculated through magnetic resonance imaging have been proposed as a useful tool to distinguish benign from malignant liver lesions. Most studies however included simple cysts in their analysis. Liver cysts are easy to diagnose, have very high ADC values and their inclusion facilitates differentiation in the ADC values between benign and malignant liver lesions groups. We prospectively evaluated the ability of ADC values to differentiate metastatic liver lesions from all benign or only solid benign liver lesions.

**Material and Methods:**

Sixty-seven adult cancer patients with 188 liver lesions were evaluated. Lesions were categorized as metastatic or benign throughout imaging and clinical evaluation. One hundred and five (105) metastatic lesions and 83 benign lesions including hemangiomas (37), cysts (42), adenomas (2) and focal nodular hyperplasias (2) were evaluated. ADC values were calculated for each lesion utilizing two b values (0 and 600 sec/mm^2^).

**Results:**

The average ADC value for cysts was 2.4×10^−3^ mm^2^/sec (CI: 2.1–2.6), for solid benign lesions was 1.4×10^−3^ mm^2^/sec (CI: 1.1–1.7) and for metastases was 1.0×10^−3^ mm^2^/sec (CI: 0.8–1.3). There was a difference between the ADC values of metastases and benign solid lesions (*p*<0.0001). With the ADC value of 1.5×10^−3^ mm^2^/sec as a cut off it is possible to distinguish metastatic from benign liver lesions, including cysts, with an accuracy of 78%. But to distinguish metastatic from benign solid liver lesions the best ADC cut off value was 1.2×10^−3^ mm^2^/sec and the accuracy drops to 71%.

**Conclusions:**

ADC values proved to be helpful in the distinction between metastasis and benign solid hepatic lesions. But the exclusion of cysts in the analysis point out to a lower cut off value and lower accuracy than previously reported.

## Introduction

The liver is a common site of hematogenous metastases. Gastrointestinal and neuroendocrine tumors as well as melanoma are the most common primary sites responsible for metastatic liver involvement [1], [2]. Ultrasound (US), computed tomography (CT) and magnetic resonance imaging (MRI) are the commonly used to detect and evaluate focal liver lesions [3]. MR of liver depends on the signal characteristics (T1 and T2 weighted signal intensities) and post-Gd imaging. The combination of these imaging techniques provides anatomic and functional imaging information to best detect and diagnose liver pathology. Recent applications of new functional methods, especially diffusion-weighted imaging (DWI), have expanded the use of MRI in the evaluation of lesions suspected for malignancy [4], [5].

Diffusion is a marker of cellularity and its quantitative analysis can be obtained through the ADC. A high ADC implies that water can move freely, indicating low cellularity and a low ADC implies that water mobility is restricted, indicating high cellularity [6]. Malignant lesions, such as liver metastases, due to the large amount of cells usually found, frequently have low ADC values. On the other hand, benign lesions such as simple cysts and hemangiomas, due to the lower amount of cells usually found, frequently have high ADC values [7].

The purpose of the present study is to evaluate the ability of apparent diffusion coefficient (ADC) to distinguish metastatic malignant from benign liver lesions.

## Materials and Methods

### Ethics statement

The ethics committee of the A. C. Camargo Cancer Center approved this study and all patients signed an informed consent form.

This prospective observational study evaluated patients admitted to our institution with focal benign and/or metastatic liver lesions from August 2010 to December 2011. The inclusion criteria in the study were: (1) adult patients over the age of 18 years; (2) patients with a histologically confirmed cancer diagnosis; (3) patients under investigation for liver lesions (4) patients not previously treated for liver metastases, and (5) patients referred to upper abdomen MRI including the DWI sequence using 0–200 and 0–600 sec/mm^2^ *b*-values (*b*: diffusion factor). A total of 67 patients with 188 lesions were included in the study.

### MRI Study

MRI exams were performed on a 1.5 T unit (GE Signa HDxt 1.5T MRI General Electric Medical Systems, Milwaukee, WI, United States) by using a body phased-array coil. Patients were in the supine position throughout the examination.

Axial T1-weighted (TR/TE of 6.3/2.7 ms; Non Fat sat) and T2-weighted \ (TR/TE  = 3200/40 ms; FS). For both T1 and T2WI the thickness of 6/0 mm, a field of view (FoV) of 30 cm and an acquisition matrix of 256×256. The T1 and T2 weighted images were used for assistance in lesion detection and characterization.

DWI was performed using triggered breathing, single-shot, echo-planar imaging (SS–EPI) sequence in the axial plane (BTR = min. 1000/max.17.000 ms; TE = min 73.2; Matrix = 192×192 mm; SI Thickness = 7.0 mm; Gap = 1.0 mm).

DWI was performed in 3 directions (X, Y and Z). Diffusion-weighted images were obtained at 0–200 sec/mm^2^ for lesion detection and 0–600 sec/mm^2^ for ADC calculation. ADC maps were automatically reconstructed for the 0–600 sec/mm^2^ *b* values diffusion-weighted images and ADC values were measured by region of interest (ROI), positioned centrally and occupying at least 50% of the lesion.

The post-contrast T1-weighted was a 3D gradient echo fat-suppressed acquisition (LAVA) performed in three dynamic phases. The arterial phase acquisition was performed with a bolus-track technique by observing the contrast entering the celiac axis, the patient was then given breathing instructions and liver images were started. The portal venous and delayed phases images were acquired at a 60 and 180 sec delay after the intravenous contrast injection. The paramagnetic contrast used was **gadoversetamide** and the dose administered was 0.1 mmol/Kg up to a maximum of 10 mmol doses using a power injector set at 2 ml/sec rate. The multiphase post-GD images were used for lesion detection and characterization.

### Lesion Characterization

Each lesion was evaluated considering the following aspects: (1) growth pattern throughout previous tests when available; (2) contours; (3) signal characteristics; (4) contrast-enhancement pattern. The liver lesions were classified as malignant or benign based on the combination of imaging features such as enhancement pattern/presence of fat, necrosis and clinical features such as the presence of new/growing liver lesion and uncontrolled systemic disease. Two radiologists with more than 10 years of experience independently reviewed the imaging and clinical data from all patients, and, when possible a final clinical diagnosis was reached by consensus.

### Data analysis

Clinical data were collected from electronic medical records. Descriptive analyses of demographic, clinical, radiological, and pathological characteristics were performed.

The variables studied are described with mean values of the absolute and relative frequency distributions, or by mean, median, minimum, and maximum values, with standard deviation provided where appropriate.

Analyses of Variance with Repeated Measurement Models [8]–[11] were employed, which consider the structure of dependency between the observers generated by a single patient due to multiple lesions.

Receiver operating characteristic (ROC) curves were performed to identify cut off values for the ADC that best classified lesions as benign and malignant (metastases) with and without the inclusion of cysts.

The free R statistical software (www.r-project.org) was used with *p*<0.05 was considered to indicate a significant difference.

## Results

### Patients

A total of 67 adult patients with 188 liver lesions met all of the inclusion criteria.

The mean age of these patients was 57 years old (range, 24–80 years), with 34 men (51%) and 33 women (49%).

### Liver lesions

From the total of 67 consecutive cancer patients that presented liver lesions on abdominal MRI, twenty-three (34.3%) patients presented with 105 liver lesions diagnosed as metastases by imaging and clinical evaluation.

Forty-four patients (65.7%) presented with 83 benign liver lesions. There were 37 (20%) hemangiomas, 42 (22%) cysts, 2 (1%) hepatic adenomas and 2 (1%) focal nodular hyperplasias (Table 1).

**Table 1:** Classification of liver lesions based on clinical data.

Diagnosis	*n*	(%)
Metastatic liver lesions	105	56
Benign lesions	83	44
- hemangioma	37	20
- cyst	42	22
- adenoma	2	1
- FNH	2	1

FNH: Focal nodular hyperplasia.

None of the patients simultaneously presented liver lesions diagnosed as benign and metastatic.

The number of liver metastases per patient ranged from 1 (one) to 15 lesions. Most patients had one to three metastatic lesions (47.4%). The mean number of lesions per patients was 1.5.

### ADC evaluation

The ADC average value for liver cysts was 2.4×10^-3^ mm^2^/sec (CI: 2.1–2.6), for solid benign lesions was 1.4×10^−3^ mm^2^/sec (CI: 1.1–1.7) and for metastases was 1.0×10^−3^ mm^2^/sec (CI: 0.8–1.3) with statistical difference (*p*<0.0001) (Table 2).

**Table 2:** ADC values (x 10^−3^ mm^2^/sec) of metastasis, benign solid lesions and cysts.

Lesion	*n*	Estimate	CI (95%)	*p*	
Metastasis	105	1.0	0.8	1.3	
Benign solid	41	1.4	1.1	1.7	<0.0001
Cyst	42	2.4	2.1	2.6	

CI: Confidence interval.

**Figure 1:**
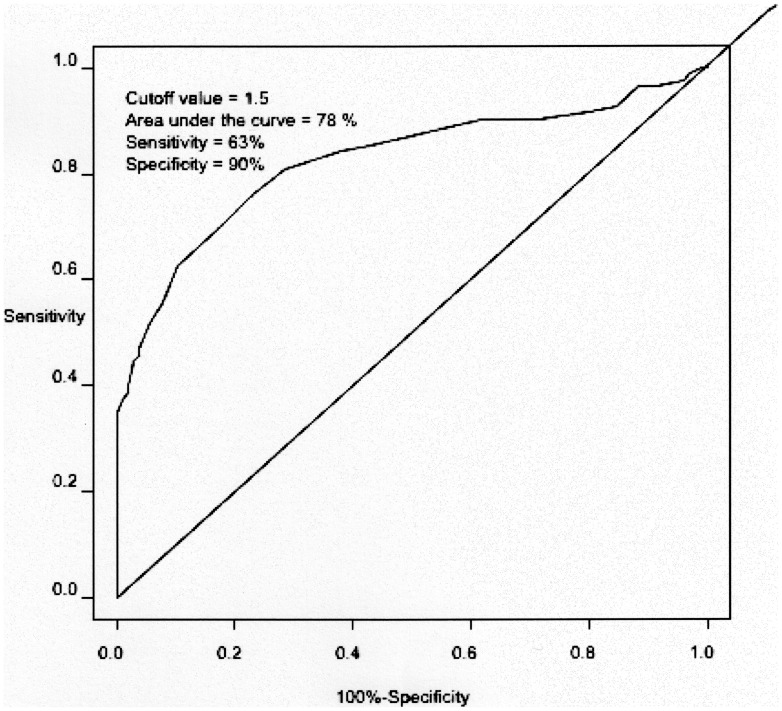
Analysis of the cutoff: metastatic liver lesions versus benign lesions, including cysts. The ROC curve generated to determine the cutoff point for the ADC value (x 10^−3^ mm^2^/sec) that best classifies liver metastases versus benign lesions. Cysts were included from this analysis.

**Figure 2:**
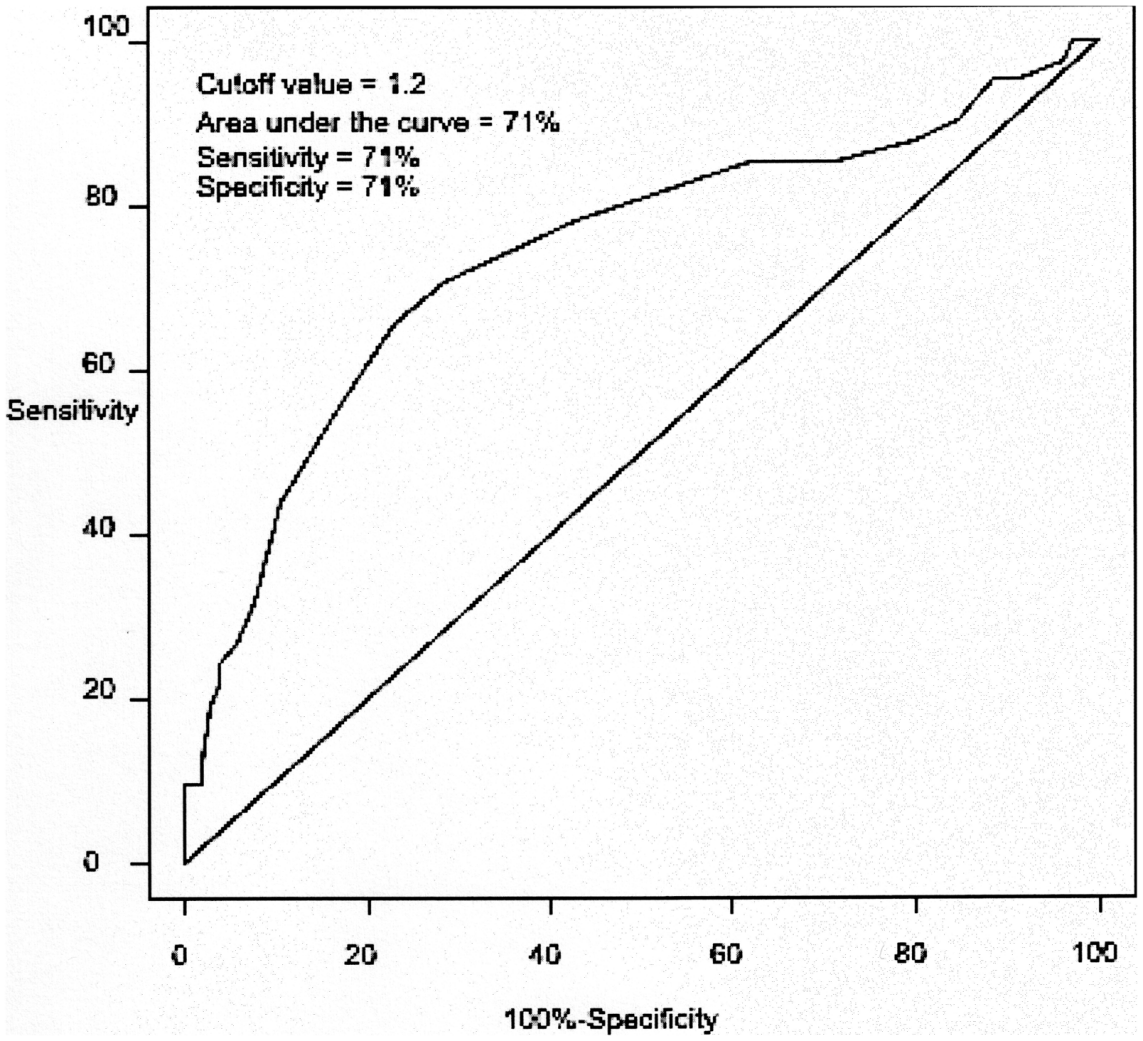
Analysis of the cutoff: metastatic liver lesions versus benign solid lesions. The ROC curve generated to determine the cutoff point for the ADC value (x 10^−3^ mm^2^/sec) that best classifies liver metastases versus solid benign lesions. Cysts were excluded from this analysis.

The cut off 1.5×10^−3^ mm^2^/sec ADC value that was best able to classify all benign lesions, including cysts, and accuracy of 78% (Figure 1). The cut off 1.2×10^−3^ mm^2^/sec ADC value that was best able to classify the solid benign lesions and the metastases, with accuracy of 71% (Figure 2).

## Discussion

A Number of technical parameters in the diffusion imaging acquisition have been pointed to potentially affect the ADC calculation such as the type of MRI equipment, the sequence parameters, apnea or free breathing technique, the number of different *b* values, and the b values utilized [12]. Low *b* values lead to an overestimation of the ADC because of the contribution of perfusion and higher *b* values underestimate the ADC because of the signal-to-noise ratio (SNR) and most authors utilize *b* values in the range of 500 to 800 sec/mm^2^ for the evaluation of focal liver lesions [12], [13].

Despite of the technique differences, reported ADC values for different focal liver lesions are quite similar between studies and around 0.9 to 1.2×10^−3^ mm^2^/sec for malignant lesions, over 1.5×10^−3^ mm^2^/sec for benign lesions and over 2.5×10^−3^ mm^2^/sec for cysts, like on a review paper of Taouli and Koh [14], on DWI in the evaluation of focal liver lesions, which reported that in malignant lesions, the ADC values ranged between 0.9 and 1.5×10^−3^ mm^2^/sec, in the benign solid lesions, the ADC values ranged between 1.4 and 2.9×10^−3^ mm^2^/sec and in cystic lesions, the ADC values ranged between 2.5 and 3.6×10^−3^ mm^2^/sec.

In the present study, in other to be in accordance to the more utilized techniques only the 0–600 sec/mm^2^ b values were employed for ADC calculations and images were obtained with triggered breathing. And the ADC values obtained for focal liver lesions were in the same range as those previously reported [14].

Prior studies proposed the use of ADC values to distinguish benign and malignant liver lesions, a common problem faced in the imaging evaluation of cancer patients. These studies report good results but they are mostly retrospective, have a small numbers of patients, and included simple cysts in the group of benign lesions [15]–[18].

On the work of review Taouli and Koh [14], all reported studies included cysts when calculating the accuracy for the differentiation of benign and malignant lesions and with similar ADC cutoff values, ranging from 1.4 to 1.6×10^−3^ mm^2^/sec they reached sensitivities of 74% to 100% and specificity of 77% to 100%.

All these studies included a relative high number of cysts leading to a selection bias due to high ADC levels encountered in these type of lesion [6], [7], [15]. In our study, the inclusion of cysts in the benign lesions group, also lead to a similar cut off ADC value of 1.5×10^−3^ mm^2^/sec for the differentiation between metastatic and benign liver lesions with a sensitivity of 63%, a specificity of 90% and an overall accuracy of 78%.

Simple cysts are usually easy to diagnose on imaging tests, especially MRI, and highly affect the statistical analysis as they have free water mobility and very high ADC values, thus raising the ADC average on the benign lesions group and consequently the cut off point, leading to a selection bias.

The differentiation between cysts and metastasis is a challenge only when dealing with primary cancers that usually provide cystic metastasis such as epithelial ovarian carcinomas or highly necrotic metastasis such as some melanomas. And here, ADC calculations may be misleading.

On a daily routine the most challenging task is to differentiate liver metastasis from benign solid liver tumors. So, for this task, the important information would be the best ADC cut off value to distinguish malignant and solid benign liver lesions and the accuracy of this differentiation.

In our data, the best ADC value cut off for the differentiation of metastatic and solid benign liver lesions was 1.2×10^−3^ mm^2^/sec with a sensitivity of 71%, a specificity of 71% and an accuracy of 71%.

And this seems to be a more appropriate approach for the use ADC values as a tool to characterize benign and metastatic liver lesions on a clinical routine. We still found a statistical significant difference in the ADC values between metastatic liver lesions and solid benign liver lesions but the cut off value and the accuracy in the differentiation seems to be lower than previously reported [7], [15], [19].

One can still utilize the lesion ADC value as a feature to be analyzed but the data should be incorporated with caution. A proper understanding of the context in which the cut off values were obtained is essential for the rational utilization of this feature in clinical practice.

A limitation of the present study should be the inclusion of patients with different primary tumors. Despite of that, differences between ADC values of these metastases were not identified in this study. The main goal of this study was to evaluate the utilization of DWI in distinguishing metastases from benign lesions in the routine of an oncology center. New studies are needed to establish the accuracy of DWI and ADC values in the characterization of focal liver lesions.

## Conclusion

The calculation of ADC is a useful tool and in combination with other imaging characteristics can help distinguish metastasis from solid benign hepatic lesions. This is very helpful in the setting when post-Gd images cannot be obtained due to poor GFR or history of allergic reaction to contrast. Ideally, ADC cut off values obtained with the exclusion of cysts in the analysis should be utilized. Since mainly solid benign lesions are sometimes difficult to distinguish from metastases.
